# Multilocus genetic risk score for diabetic retinopathy in the Han Chinese population of Taiwan

**DOI:** 10.1038/s41598-018-32916-y

**Published:** 2018-09-28

**Authors:** Wen-Ling Liao, Jang-Ming Lin, Wen-Lu Chen, Ming-Chia Hsieh, Chia-Ming Wu, Ya-Wen Chang, Yu-Chuen Huang, Fuu-Jen Tsai

**Affiliations:** 1 Graduate Institute of Integrated Medicine, China Medical University, Taichung, 404 Taiwan; 2 Center for Personalized Medicine, China Medical University Hospital, Taichung, 404 Taiwan; 3 Department of Ophthalmology, China Medical University Hospital, Taichung, 404 Taiwan; 4 Division of Endocrinology and Metabolism, Department of Internal Medicine, Changhua Christian Hospital, Changhua, Taiwan; 5 Human Genetic Center, Department of Medical Research, China Medical University Hospital, China Medical University, Taichung, 404 Taiwan; 6 School of Chinese Medicine, China Medical University, Taichung, 404 Taiwan; 7 Department of Health and Nutrition Biotechnology, Asia University, Taichung, 413 Taiwan

## Abstract

The aim of this study is to explore the effect of genetic variation on diabetic retinopathy (DR) risk in a Taiwanese population. The logistic regression model was used to evaluate the relationship between DR status and risk factors, including the conventional parameters and genetic risk score (GRS). Candidate single nucleotide polymorphisms (SNPs) in GRS were selected based on previous reports with a combined *P* < 10^−4^ (genome-wide association) and *P* < 0.05 (meta-analysis). In total, 58 SNPs in 44 susceptibility loci were selected, and four were used to calculate GRS. After adjustment for age, systolic blood pressure, diabetes duration, and HbA1c, the DR risk was 4.95 times higher for patients in the top GRS third tile than for those in the bottom third tile (95% CI = 2.99–8.18; *P* < 0.001). The addition of genetic information improved DR prediction, increasing the area under the curve (AUC) from 0.72 to 0.77 (*P* = 0.0024) and improving the sensitivity of the model such that 40 more subjects were reclassified into DR status. The developed multivariate logistic regression model combining conventional risk factors and the multilocus GRS can predict DR, thus enabling timely treatment to reduce blindness in T2D patients.

## Introduction

Diabetic retinopathy (DR) is a common microvascular complication of diabetes and a leading cause of blindness in adults^1,2^. The prevalence of DR among type 2 diabetes (T2D) patients is about 22%, and up to 80% of patients suffering from diabetes for over 10 years develop DR. The condition progresses from non-proliferative DR (NPDR) to proliferative DR (PDR) and early detection of NPDR may lead to a 60% reduction in PDR and 83% reduction in blindness^3^. However, many diabetic patients with mild NPDR are not aware of the condition because it does not usually affect their vision, and, since NPDR does not require treatment, it is difficult to prevent its progression to PDR. As DR is a consequence of diabetes, patients should take general precautions to improve their blood sugar control in order to stop or slow the disease progression, but there are no reliable biomarkers for predicting NPDR and/or its development into PDR. Therefore, it is important to identify risk factors for DR progression, which would enable implementation of timely and effective treatment to reduce blindness in T2D patients.

Previous studies showed that a number of traditional risk factors such as age, gender, diabetes duration, fasting plasma glucose, glycosylated hemoglobin (HbA1c), and systolic blood pressure (SBP) could predict the progression and severity of DR^4–7^. Multifocal electroretinogram (mfERG) with implicit time (IT) and Z-score, foveal thickness, and blood biomarkers, including lipid components (high- and low-density lipoprotein [HDL and LDL, respectively], cholesterol, and triglycerides [TGs]), apolipoprotein, advanced glycation end products, and cytokines could also be used to predict the DR status^8–11^. Furthermore, it is established that heredity plays a key role in the pathogenesis of diabetes and its complications^12–14^, and familial clustering of DR among T2D patients suggests strong contribution of genetic factors to the risk of developing the disease^13^.

A number of genome-wide association studies (GWASs) and candidate gene association studies have been performed to examine genetic susceptibility to DR in different ethnic populations^15–17^. However, a comprehensive composite model that can estimate the combined effect of conventional risk factors and genetic background to predict the occurrence of DR in diabetic patients is limited. Therefore, the aim of this study was to investigate the association between previously reported genetic variants and DR risk, and develop a multifactorial logistic regression model to predict DR in the Han Chinese population of Taiwan.

## Results

### Characteristics of the study participants

Among the 1,055 T2D patients enrolled in this replication study, 468 had DR (case) and 587 did not have DR (non-DR, control). The male to female ratios were 1.03 (238/230) for the DR group and 1.13 (311/276) for the control group, indicating no statistically significant difference in gender distribution (*P* = 0.492 by Pearson chi-square test). However, age, diabetes duration, age of onset, HbA1c, SBP, diastolic blood pressure (DBP) in the DR group were significantly higher compared to control (*P* < 0.001 for all parameters; Table 1). Additionally, DR groups had lower eGFR value and high ACR value in limited subjects (n = 564 for control and 162 for DR).

**Table 1:** Demographics of the study population.

	T2D control (N = 587)	DR (N = 468)	OR	*P* value
Gender				
Male	311 (53.0%)	238 (50.9%)	Ref.	Ref.
Female	276 (47.0%)	230 (49.1%)	1.09	0.492
Age (years)				
<55	229 (39.0)	100 (21.4)	Ref.	Ref.
55–65	191 (32.5)	199 (42.5)	2.39	<0.001*
>65	167 (28.4)	169 (36.1)	2.32	<0.001*
DM duration(years)				
≦10	420 (71.6)	177 (38.6)	Ref.	Ref.
>10	167 (28.4)	281 (61.4)	3.99	<0.001*
Age of onset (years)				
<45	161 (27.4)	173 (37.8)	Ref.	Ref.
45–55	234 (39.9)	172 (37.6)	0.68	0.011*
>55	192 (32.7)	113 (24.7)	0.55	<0.001*
HbA1c				
≦8	395 (67.3)	234 (50.0)	Ref.	Ref.
>8	192 (32.7)	234 (50.0)	2.06	<0.001*
SBP				
<140	316 (58.8)	177 (38.8)	Ref.	Ref.
≥140	221 (41.2)	279 (61.2)	2.25	<0.001*
DBP				
<90	459 (85.5)	358 (78.5)	Ref.	Ref.
≥90	78 (14.5)	98 (21.5)	1.61	0.004*
Fasting glucose^#^				
<126	202 (34.7)	95 (35.1)	Ref.	Ref.
126–155	216 (37.1)	71 (26.2)	0.70	0.053
>155	164 (28.2)	105 (38.7)	1.36	0.080
HDL^#^				
<41	198 (34.5)	59 (33.9)	Ref.	Ref.
41–52	194 (33.8)	52 (29.9)	0.90	0.62
>52	182 (31.7)	63 (36.2)	1.16	0.47
LDL^#^				
<103	204 (35.5)	62 (35.6)	Ref.	Ref.
103–132	189 (32.9)	65 (37.4)	1.13	0.55
>132	182 (31.7)	47 (27.0)	0.85	0.46
TG^#^				
<103	182 (31.8)	57 (32.9)	Ref.	Ref.
103–171	194 (33.9)	57 (32.9)	0.94	0.77
>171	196 (34.3)	59 (34.1)	0.96	0.85
eGFR^#^				
>90	372 (64.7)	80 (46.0)	Ref.	Ref.
60–90	154 (26.8)	61 (35.1)	1.84	0.002*
<60	49 (8.5)	33 (19.0)	3.13	<0.001*
ACR^#^				
<30	398 (70.6)	68 (42.0)	Ref.	Ref.
30–300	135 (23.9)	69 (42.6)	2.99	<0.001*
>300	31 (5.5)	25 (15.4)	4.72	<0.001*

Values are presented as N (%).

Abbreviation: T2D, type 2 diabetes; DR, diabetic retinopathy; DM duration: diabetes mellitus duration; HbA1c: hemoglobin A1c; SBP/DBP: systolic/diastolic blood pressure; HDL: high density lipoprotein; LDL: low density lipoprotein; TG: triglyceride; eGFR: estimated glomerular filtration rate; ACR: urine albumin creatinine ratio; Ref., reference.

^#^Results from limited subjects (N = 564 and 162 for T2D control and DR, respectively). *Represent *P* value less than 0.05.

### Association between individual single nucleotide polymorphisms and DR risk

Among the 58 susceptibility single nucleotide polymorphisms (SNPs), the majority (except rs4762 and rs5498) passed the Hardy-Weinberg equilibrium (HWE) test (*P* < 0.05). For most of the passed SNPs (except rs1801282, rs487083, rs7903146, rs10501943, rs3742872, and rs13306430), minor allele frequency (MAF) exceeded 5% and were similar to those in Han Chinese from Beijing (NCBI GRCh37.p13 assembly) according to the dbSNP website (based on 1000 Genomes project; https://www.ncbi.nlm.nih.gov/variation/tools/1000genomes/) (Table S1). None of the 58 susceptibility SNPs showed statistical significance in the additive model after Bonferroni correction.

Then, we genotyped/imputed all SNPs located in 44 susceptibility genes; among them, 93 had *P* < 0.05 in the additive model (Table S2). After applying all selection criteria, including *P* ≥ 0.05 in the HWE test, MAF > 0.05, imputation info ≥0.4, and *P* < 5.38 × 10^−4^ after Bonferroni correction, four SNPs, rs4748644, rs11101385, rs61893374, and rs142644390, were proved significant in the additive models (*P* = 3.81E-04, 1.11E-05, 2.73E-06, and 1.63E-05, respectively). The identified SNPs are located in the *PLXDC2* (Plexin domain-containing 2)*, ARHGAP22* (Rho GTPase-activating Protein 22), *CNTN5* (Contactin 5), and *FMN1* (Formin 1) genes, respectively (Table 2).

**Table 2:** Association between genetic SNPs and DR status among Taiwanese population.

rs ID	Gene	Chr.	Risk allele	Homozygous dominant/Heterozygous/Homozygous recessive genotype frequency		Additive model	
				T2D control (N = 294)	DR cases (N = 234)	OR (95%CI)	*P*
rs4748644	PLXDC2	10	T	64/153/77	84/105/45	1.64 (1.25–2.16)	3.81E-04
rs11101385	ARHGAP22	10	A	257/35/2	167/6/61	2.69 (1.73–4.19)	1.11E-05
rs61893374	CNTN5	11	T	255/36/0	171/57/6	3.15 (1.95–5.10)	2.73E-06
rs142644390	FMN1	15	A	213/75/6	206/27/1	2.93 (1.80–4.78)	1.63E-05

Abbreviations: SNP, single nucleotide polymorphism; Chr., chromosome; DR, diabetic retinopathy; OR, odds ratio; CI, confidence interval.

OR calculation was conducted according to the defined risk alleles (Var/Ref).

### Cumulative effect of the four genetic loci on the DR risk

To build the model, we first calculated the multiplex genetic risk score (GRS) for each individual. The cumulative effect of the four significant SNPs was assessed by counting the number of risk genotypes in each individual, and the weighted GRS was calculated based on the logarithm odd ratio (OR) of the susceptibility SNPs. The mean number of risk alleles was 3.24 ± 1.07 (range 0–6), and the mean weighted GRS was 3.22 ± 0.99 (range 0–6.32) in the derivation sample. The distribution of risk alleles and weighted GRS is shown in Figure S1. All patients were divided into three groups based on the number of risk alleles. The data indicated that the DR risk increased with the number of risk genotypes (*P* = 5.98 × 10^−12^; Cochran-Armitage Trend test). Compared with individuals in the lowest range of weighted GRS, the ORs with 95% confidence intervals (CIs) for those in the middle and high range were 2.14 (1.22–3.74) and 4.95 (2.99–8.18), respectively (Table 3) in the derivation sample. These results suggest a cumulative effect of the four SNPs on the DR risk.

**Table 3:** Multivariate association between conventional, genetic, and diabetic retinopathy.

	Overall Sample			Derivation Sample			Test Sample		
	OR	95% CI	*P* value	OR	95% CI	*P* value	OR	95% CI	*P* value
Age (years)	1.00	0.99–1.02	0.660	1.00	0.98–1.03	0.758	1.00	0.98–1.03	0.732
Duration (years)									
≦10	Ref.	Ref.	Ref.	Ref.	Ref.	Ref.	Ref.	Ref.	Ref.
>10	3.49	2.58–4.71	3.69 × 10^−16^	3.78	2.43–5.87	3.48 × 10^−9^	3.39	2.22–5.15	1.29 × 10^−8^
HbA1c (%)									
≦8	Ref.	Ref.	Ref.	Ref.	Ref.	Ref.	Ref.	Ref.	Ref.
>8	1.90	1.43–2.53	1.10 × 10^−5^	1.64	1.09–2.49	0.018	2.20	1.47–3.30	1.41 × 10^−4^
SBP (mmHg)									
<140	Ref.	Ref.	Ref.	Ref.	Ref.	Ref.	Ref.	Ref.	Ref.
≧140	2.04	1.54–2.72	9.20 × 10^−7^	2.19	1.45–3.32	2.15 × 10^−4^	1.95	1.30–2.91	1.16 × 10^−3^
GRS									
Q1 (<3.01)	Ref.	Ref.	Ref.	Ref.	Ref.	Ref.	Ref.	Ref.	Ref.
Q2 (3.01 to 3.53)	1.30	0.89–1.91	0.174	2.14	1.22–3.74	0.008	0.82	0.48–1.41	0.471
Q3 (>3.53)	2.75	1.97–3.84	3.20 × 10^−9^	4.95	2.99–8.18	4.67 × 10^−10^	1.63	1.03–2.58	0.038
*P* for trend			7.93 × 10^−12^			5.98 × 10^−12^			5.93 × 10^−3^

Abbreviation: T2D, type 2 diabetes; DR, diabetic retinopathy; OR, odds ratio; CI, confidence interval; SBP, systolic blood pressure; GRS, Genetic risk score; Q, quantile; Ref., reference. The genetic risk score was calculated based on 4 SNPs. The respective risk genotypes were shown in Table 2.

### Compared models with and without genetic markers in the derivation sample

The best model retained four “conventional” variables (age, HbA1c, diabetes duration, and SBP) and the weighted GRS (computed based on the four SNPs) as significant independent variables (Table 3). The total risk score of the DR risk was calculated for each subject based on the regression coefficients of all variables (Table S3). The sensitivity was 74.9% (167/223) and specificity was 66.2% (178/269) when the optimal cutoff identified for risk score was ≥2.28 as screen positive and <2.28 as screen negative.

**Figure 1:**
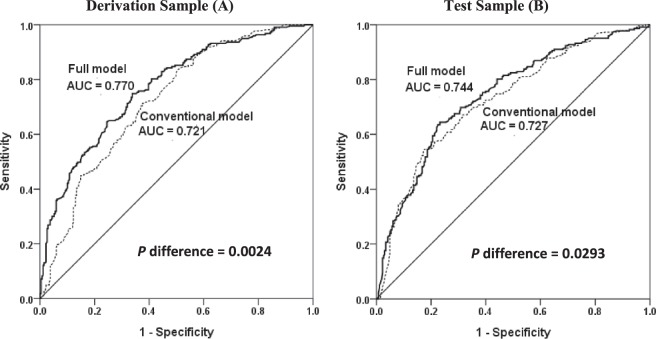
Receiver Operating Characteristic (ROC) curve and area under the curve (AUC) in the Taiwanese population. ROC curves and AUC of the models built for derivation sample (**A**) and test sample (**B**) (AUC = 0.770 and 0.744, respectively). The diagonal line indicates zero predictive value of the model.

The probability of DR for each patient was calculated using the equation:

$${\rm{P}}(\mathrm{DR}=1)={\rm{e}}{\rm{\alpha }}+{\rm{\beta }}^{\prime} {\rm{X}}/(1+{\rm{e}}{\rm{\alpha }}+{\rm{\beta }}^{\prime} {\rm{X}}),$$

where α + β′X = −2.501 + 0.003 × Age + 0.497 × 1(HbA1c = 2) + 1.329 × 1(duration = 2) + 0.783 × 1(SBP = 2) + 0.759 × 1(weighted GRS = 2) + 1.599 × 1(weighted GRS = 3). The area under the curve (AUC) value was 0.77 (95% CI = 0.729–0.811), indicating that the model had a reasonably good discrimination ability. To determine the impact of the genetic factors on the model, a conventional model that included only age, diabetes duration, HbA1c, and SBP was built and the AUC value became 0.721 (95% CI = 0.677, 0.766). Comparison of the best and conventional models revealed a significant difference between the two models, indicating that the incorporation of the genetic data improved DR prediction compared to the conventional model (*P* = 2.4 × 10^−3^) (Fig. 1A). The total correct classification rate was 65.2% in the conventional model and 69.5% in the best model containing both conventional and genetic variables. The sensitivity of the model was increased such that 40 more DR subjects were reclassified into DR status after the inclusion of the genetic variables into the model (sensitivity = 47.1% vs. 65.0% in conventional and best models, respectively). However, the specificity of the model was decreased (19 non-DR subjects were miss-classified into DR groups) (specificity = 80.3% vs. 73.2% in conventional and best model, respectively).

### Assessment of the discriminatory ability of models in different datasets

To assess the discriminatory ability of the models, the model obtained from the derivation sample was applied to the test sample and validation sample and the AUC was determined for all datasets. The model showed a similar discrimination ability in the test sample (AUC = 0.744 [95% C.I. = 0.700, 0.787] (Fig. 1B)) compared to in the derivation dataset (AUC = 0.77 (95% CI = 0.729–0.811); *P* = 0.3926). However, the AUC value decreased when the best model was applied to the validation sample (AUC = 0.662 [95% CI = 0.601, 0.722]), and the difference between the two AUC values was significant (*P* = 0.0039) (Figure S2).

We also calculated positive and negative predictive values using the same risk score cutoff of 2.28 derived from derivation samples for test and validation samples. For the test set, the positive predictive value (PPV) was 72.0% (118 individuals with a risk score ≥2.28, of whom 85 were DR subjects) and the negative predictive value (NPV) was 62.7% (367 individuals with risk score <2.28, of whom 230 were non DR subjects). The corresponding values for the validation sample were PPV of 31.3% (32 individuals with a risk score ≥2.28, of whom 10 were DR subjects) and a NPV of 83.4% (494 individuals with risk score <2.28, of whom 412 were non DR subjects).

## Discussion

In the present study, we investigated the DR risk among the Taiwanese population according to genetic variants identified by GWASs and meta-analysis, and built a prediction model. In the 44 replicated genes, four SNPs were identified to be associated with the DR risk in our population. Furthermore, a GRS based on the number of risk alleles from these four SNPs was calculated for each individual, and an independently cumulative genetic effect on the DR risk was observed in the multivariate models after adjusting for diabetes duration, HbA1c, and SBP. The addition of the genetic information to the conventional variables improved the discrimination ability for DR, increasing the AUC from 0.721 to 0.770. Furthermore, the sensitivity of model was increased after addition of GRS, which indicated that the model with genetic markers may be a clinical useful improvement, thus enabling timely treatment to reduce blindness in T2D patients.

Previous studies have identified genetic variants significantly associated with DR in various ethnic groups^17–21^. Here, we confirmed the association of *PLXDC2, ARHGAP22, CNTN5*, and *FMN1* polymorphisms with DR in the Taiwanese population. The four identified SNPs were not linked to previously reported SNPs in the same loci and differed from them in allele frequencies. *PLXDC2* and *ARHGAP22* were earlier identified as DR risk genes by GWAS^19^ and meta-analysis^20^. SNPs rs1571942 and rs12219125 in the *PLXDC2* locus were first reported as risk factors for T2D patients with retinopathy^19^ and showed nominally significant (*P* < 0.05) association with severe DR (≥severe NPDR or history of panretinal photocoagulation) for T1D patients. In the present study, rs4748644 was not linked to rs1571942 and rs12219125 (r^2^ = 0.045 for both; Table S4) and showed a different MAF (48.50% vs. 12.1% and 12.1%, respectively). SNPs rs4838605^17,19^, rs11101355^19^, and rs11101357^19^ in the *ARHGAP22* gene were identified as associated with DR in Han Chinese and Caucasian patients with both T1D and T2D. Rho GTPase-activating protein encoded by the *ARHGAP22* gene is known to be involved in insulin response mechanisms regulating endothelial cell migration and cancer metastasis^22^. In the present study, rs11101385 was highly linked to the reported *ARHGAP22* SNPs (rs4838605, rs11101355, and rs11101357; r^2^ = 0.712, 0.8, and 0.8, respectively; Table S4), with MAF ranging from 10.24% to 16.16% in the Taiwanese population. A previous GWAS identified *CNTN5* and *FMN1* as DR susceptibility genes among Mexican Americans: rs10501943 (*P* = 2.53 × 10^−4^) mapped to *CNTN5* intron regions on chromosome 11q22 and rs10519765 (*P* = 6.21 × 10^−5^) mapped to *FMN1* intron regions on chromosome 15q13 were found associated with severe DR^18^. In the present study, we identified other SNPs, rs61893374 in the *CNTN5* gene and rs142644390 in the *FMN1* gene as associated with DR in the Taiwanese population. These SNPs showed low linkage with those reported for Mexican Americans (r^2^ = 0.007 for rs61893374 vs. rs10501943 in *CNTN5* and r^2^ = 0.003 for rs142644390 vs. rs10519765 in *FMN1*; Table S4) and had different MAFs (11.7% for rs61893374 vs. 4.9% for rs10501943 in *CNTN5*, and 11.2% for rs142644390 vs. 8.3% for rs10519765 in *FMN1*)^18^. These results indicate that the selected genes with critical biological functions play an important role in DR development in the Taiwanese population.

Our study has both strengths and limitations. In previous GWASs on DR, none of the regions reached genome-wide statistical significance^18,19,23^. The limitations of those studies include modest sample size by GWAS standards, heterogeneous phenotypes (PDR, NPDR, and diabetic macular edema), and poor DR standardization. Our study has several strengths. First, all participants, who were unrelated Han Chinese from Taiwan, underwent exhaustive ophthalmological examination following detailed protocols for both non-DR and DR patients, which limited chances of misclassification. Second, the genetic markers we selected were based on previous studies and all the genetic markers have been reported to be relevant to mechanisms of T2D or DR development. Therefore, these genetic markers represented robust and replicated variants for DR. Third, the difference in allelic frequencies for the four SNPs between the two groups ranged from 7.6% to 20.7% (sample size, 587 vs. 468 for T2D controls and DR cases, respectively) in the current study, thereby raising the statistical power for selecting the appropriate genetic markers of DR risk to over 96%.

We also recognized several limitations in the present study. First, not all of the suggested risk factors such as mfERG, foveal thickness, and certain blood biomarkers (cytokines) could be evaluated in the present study because the relevant information was not collected at the beginning of the study. Second, the cross sectional study design was used in current study. It is necessary to conduct a long-term follow-up evaluation of non-DR T2D patients who carry the risk genotypes to determine DR susceptibility depending on the identified SNPs and prediction model. Third, the AUC value of the model and PPV in the validation sample was decreased compared to that in the derivation sample. Additionally, inconsistency of the association for the genetic variants chosen for GRS was observed in the validation sample. This could be due to several reasons, including the small sample size, the self-reported DR status, and the fact that more participants had diabetic nephropathy (eGFR <90 mL/min/1.73 m^2^) in the validation sample.

In conclusion, we analyzed the association between a panel of genetic variants and the DR risk and developed a multivariate logistic regression model to predict DR in the Taiwanese population. Confirmatory studies in a cohort of a larger size should be performed in the near future to validate our model.

## Methods

### Study participants for derivation population

The study involved 1,055 T2D patients 20 years and older, who were recruited from the China Medical University Hospital (CMUH), Taichung, Taiwan. Diabetes was diagnosed based on medical records and fasting plasma glucose levels according to the American Diabetes Association Criteria^24^. Patients with type 1 diabetes (T1D), gestational diabetes, and maturity onset diabetes of the young (MODY) were excluded from this study. The participants were of Han Chinese ethnicity characteristic for 98% of the population in Taiwan. All T2D patients underwent complete ophthalmologic testing, including corrected visual acuity, fundoscopic examination, and fundus photography. An expert ophthalmologist graded DR according to the International Clinical DR Disease Severity Scale proposed by the American Academy of Ophthalmology^25^. The whole groups of subjects were randomly assigned to a derivation set (n = 528) and a test set (n = 527) at a 1:1 ratio. The two databases were found to be compatible (Table S5).

### Study participants for validation

Another group of 542 T2D patients from three different hospitals, including CMUH, ChangHua Christian Hospital (CCH), and Taiwan Biobank, were selected for validation. The DR status was self-reported by the subjects. The characteristics of 95 DR and 447 non-DR patients are presented in Table S6.

The study was approved by the CMUH and CCH Institutional Review Board and informed consent was obtained from all participants. We confirm that all experiments of the study were performed in accordance with relevant guidelines and regulations.

### Power calculation

With regard to the sample size, we estimated that for a type 1 error of 5% and a power of 80%, a total of 511 participants would be needed to detect an OR of 2.0 for a dependent and an independent variable both with a prevalence of 0.2. Thus, the sample size was sufficient to test small effect sizes given that the prevalence of the corresponding DR was not too low. G*Power Version 3.1.7^26,27^ uses the algorithm described by Hsieh *et al*^28^. Table S7 was presented the sample size calculation based on different conditions.

### Data collection

Data regarding age, gender, age at T2D diagnosis, and ocular history were collected from questionnaires. For each patient, SBP and DBP, waist and hip circumferences, and body mass index were determined, and blood samples were collected by venipuncture for genomic DNA isolation and serological tests, including fasting glucose and HbA1c, at the time of enrollment in the study.

### Genetic marker selection and genotyping/imputation

All of the genetic markers were selected based on previous reports. The inclusion criteria of candidate SNPs were set as a combined *P* < 10^−4^ for GWAS^18,19,23,29^ and a *P* < 0.05 for meta-analysis^20,21,30–50^. A total of 58 SNPs in 44 susceptibility loci were evaluated in our study (details are summarized in Table S1). Furthermore, we performed genotyping or imputation for all the SNPs in these 44 susceptibility loci using Illumina HumanHap550-Duo BeadChips or Affymetrix-TWB chips. Genotypic data were quality-controlled, and SNPs were excluded from further analysis if: (1) MAF was less than 5% in non-DR T2D controls, (2) the total call rate was less than 95% for both DR and control patients, or (3) an SNP significantly departed from HWE proportions for controls (*P* < 0.05). For the untyped SNPs, genotype imputation was performed according to the methodology of Howie *et al*.^51^ implemented in impute v2 (http://mathgen.stats.ox.ac.uk/impute/impute_v2.html). The panel from 1,000 Genomes Project was used as reference for imputation, and the software chose the best customized reference set for each individual. SNPs with low imputation quality (info < 0.4) and those in the same gene showing strong disequilibrium with each other (D′ >0.8) were excluded from further analysis. Each SNP was tested for association with DR in an additive model by multivariable logistic regression analysis after adjustment for T2D duration and HbA1c level. The genotypes were coded in the additive model as “0” for non-risk allele homozygotes, “1” for heterozygotes, and “2” for risk allele homozygotes. A total of 93 SNPs in 24 genetic loci were identified (Table S2); among them, four showed statistical significance after Bonferroni correction (cut-off *P* value for Bonferroni correction = 0.05/93 = 5.38 × 10^−4^). The flow chart for genetic marker selection is presented in Figure S3.

### Statistical Analysis

Analyses were performed using the logistic regression model to evaluate the relationship between DR status and the following of two groups of factors: conventional parameters and genetic markers. These factors were selected based on literature review and information collected from databases. Conventional parameters included age, sex, DM duration, SBP, DBP, and serological markers (such as fasting plasma glucose, HbA1c, and lipid markers (HDL, LDL, cholesterol, and TGs). For the genetic markers, the four significant SNPs that passed the selection process described above were used to calculate the weighted GRS. The GRS for each individual was calculated based on the number of risk alleles weighted by the effect size (logarithm of ORs) according to the following equation^52^: weighted GRS = 4/3.462 × [(*ARHGAP22*_rs11101385_Risk × 0.932) + (*FMN1*_rs142644390_Risk × 1.077) + (*PLXDC2*_rs4748644_Risk × 0.451) + (*CNTN5*_rs61893374_Risk × 1.002)]. The weighted GRSs were divided into three equal groups to calculate the cumulative effect. The weighted GRS in the prediction model was calculated from the derivation sample and applied to test/validation samples.

The ORs with 95% CIs of predictor variables were estimated using logistic regression models to develop a best model of DR risk in the derivation sample and to assess the model’s discrimination ability in the test sample and validation sample. We used three steps to select independent variables that result in a “best” model^53,54^. First, we conducted a univariable analysis of each variable. Second, we selected variables *P* < 0.05 as a candidate in the multivariable model. Third, we constructed a multivariable model with candidate variables by the backward selection method. To determine the impact of genetic factors, two models were compared, including the model with “conventional” variables (age, diabetes duration, HbA1c, and SBP) and the model with conventional and GRS. The difference between AUC values from models was evaluated by Z statistics^55^.

Furthermore, the total risk score of the DR risk was calculated for each subject based on the regression coefficients of all conventional parameters and GRS in the best model^56–58^. Receiver operating characteristic (ROC) curves were generated to quantify the predictive accuracy of the models, and the AUC was used to assess the discriminatory ability of the models. To assess discrimination, the model obtained from the derivation sample was applied to the test sample and validation sample and the AUC was determined for all samples. All statistical analyses were performed using SPSS software, v. 12.0 for Windows (IBM, Armonk, NY, USA).

## Electronic supplementary material

Supplemental information
