# Supplementary material for: Multilocus genetic risk score for diabetic retinopathy in the Han Chinese population of Taiwan
Source: Sci Rep. 2018 Sep 28;8:14535. doi: 10.1038/s41598-018-32916-y (PMC6162301; doi:10.1038/s41598-018-32916-y)
Supplement: Supplementary file 1 — Supplemental information [file 41598_2018_32916_MOESM1_ESM.pdf]

Multilocus genetic risk score for diabetic retinopathy in the Han Chinese population of Taiwan

Wen-Ling Liao DrPH[1,2], Jang-Ming Lin MD[3], Wen-Lu Chen MD[3], Ming-Chia Hsieh MD[1,4], Chia-Ming Wu MS[5], Ya-Wen Chang MS[5], Yu-Chuen Huang PhD[5,6,+], Fuu-Jen Tsai MD, PhD[5,6,7,+,*]

[1]Graduate Institute of Integrated Medicine, China Medical University, Taichung, 404, Taiwan
[2]Center for Personalized Medicine, China Medical University Hospital, Taichung, 404, Taiwan
[3]Department of Ophthalmology, China Medical University Hospital, Taichung, 404, Taiwan
[4]Division of Endocrinology and Metabolism, Department of Internal Medicine, Changhua Christian Hospital, Changhua, Taiwan
[5]Human Genetic Center, Department of Medical Research, China Medical University Hospital, China Medical University, Taichung, 404, Taiwan
[6]School of Chinese Medicine, China Medical University, Taichung, 404, Taiwan
[7]Department of Health and Nutrition Biotechnology, Asia University, Taichung, 413, Taiwan
*corresponding. F.J.T (d0704@mail.cmuh.org.tw).
[+]FJT and YCH contributed equally to this work.

Table S1. Reference for genetic loci identified from GWAS or meta-analysis of diabetic retinopathy

No.	rs ID	Gene	Chr.	Position	Minor Allele	MAF (%) in CHB[a]	MAF (%) in controls	Info[b] (Chip1[c]/Chip2[d])	*P* for HWE in controls	Reference
1	rs1801133	MTHFR	1	11856378	A	46.6%	25.51%	— / —	0.567	2011 Huang[1];2012 Niu[2]; 2016 Luo[3]
2	rs12092121	MYSM1	1	59153010	G	40.3%	36.22%	— / 1.00	0.248	2011 Huang[1]
3	rs2811893	MYSM1	1	59162148	C	40.3%	36.22%	— / —	0.248	2011 Huang[1]
4	rs6427247	SCYl1BP1	1	170380480	G	37.9%	26.53%	1.00 / 0.91	0.696	2010 Fu[4]
5	rs4762	AGT	1	230845977	A	9.7%	10.24%	— / 0.98	0.013	2009 Abhary[5]
6	rs699549	LOC727982	2	4705263	T	30.1%	23.81%	1.00 / 0.99	0.164	2010 Fu[4]
7	rs763970	HNMT	2	138636133	A	23.8%	30.44%	0.99 / 0.99	0.149	2010 Fu[4]
8	rs1399634	LRP2-BBS5	2	170244607	A	38.4%	38.91%	0.96 / 0.97	0.930	2013 Sheu[6]
9	rs2380261	ARL4C-SH3BP4	2	235641180	A	34.5%	39.63%	0.99 / 0.98	0.441	2013 Sheu[6]
10	rs1801282	PPARgmma2	3	12393125	G	4.9%	3.06%	0.93 / —	0.588	2012 Ma[7]
11	rs1197310	BFSP2	3	133128224	A	53.9%	51.19%	0.97 / 0.66	0.239	2010 Fu[4]
12	rs4470583	FSTL5	4	162250932	A	5.8%	8.33%	1.00 / 1.00	0.119	2011 Huang[1]
13	rs2910964	ITGA2	5	52350521	A	28.6%	28.57%	1.00 / 1.00	0.568	2009 Abhary[5]
14	rs1445754	EDIL3	5	83575631	T	3.9%	5.10%	0.95 / 0.99	0.777	2010 Fu[4]
15	rs13163610	KIAA0825	5	93548877	C	6.3%	5.82%	— / 0.98	0.991	2011 Huang[1]
16	rs17376456	KIAA0825	5	93557702	G	6.8%	5.61%	— / —	0.308	2011 Huang[1]
17	rs2300782	CAMK4	5	110788785	T	51.9%	44.56%	0.98 / 0.98	0.118	2010 Fu[4]
18	rs1800629	TNF-alpha	6	31543031	A	9.2%	11.77%	0.99 / —	0.972	2014 Meng[8]
19	rs184003	RAGE	6	32150296	A	18.5%	20.41%	0.98 / 0.96	0.244	2012 Niu[9]; 2012 Yuan[10]
20	rs2070600	RAGE	6	32151443	T	22.3%	22.96%	— / —	0.409	2012 Niu[9]; 2012 Yuan[10]

21	rs1800624	RAGE	6	32152387	T	14.6%	9.18%	0.99 / 0.95	0.288	2012 Niu[9]; 2012 Yuan[10]
22	rs1800625	RAGE	6	32152442	G	12.1%	7.14%	0.99 / 0.94	0.187	2012 Niu[9]; 2012 Yuan[10]
23	rs1224329	TMEM217	6	37180237	G	9.2%	11.56%	— / 0.9	0.271	2013 Lin[11]
24	rs1150790	TMEM217	6	37180523	A	9.2%	11.56%	— / 0.91	0.271	2013 Lin[11]
25	rs833061	VEGF	6	43737486	C	27.2%	26.87%	0.99 / 1.00	0.819	2013 Qiu[12];2014 Han[13]
26	rs2010963	VEGF	6	43738350	C	44.7%	37.24%	0.98 / 0.99	0.580	2013 Qiu[12];2014 Han[13]
27	rs2146323	VEGF	6	43745095	A	24.8%	25.85%	0.92 / 0.93	0.616	2013 Qiu[12];2014 Han[13];2015 Zeng[14]
28	rs3025039	VEGF	6	43752536	T	18.5%	14.46%	0.81 / —	0.069	2013 Qiu[12];2014 Han[13]
29	rs713050	MRPL14	6	44092129	G	7.3%	5.27%	— / —	0.340	2013 Lin[11]
30	rs487083	GRIK2	6	102026932	G	3.9%	3.40%	— / 0.97	0.546	2013 Lin[11]
31	rs4880	SOD2	6	160113872	G	11.7%	13.61%	0.97 / —	0.826	2011 Tian[15]
32	rs39059	CHN2	7	29255470	G	39.3%	37.24%	0.99 / 1.00	0.422	2011 Hu[16];2015 Hosseini[17]
33	rs1799768	SERPINE1(PAI-1)	7	100769706	TG	43.2%	43.69%	0.92 / 0.89	0.464	2009 Abhary[5]
34	rs759853	AKR1B1	7	134143958	A	18.0%	18.03%	0.99 / 0.94	0.827	2009 Abhary[5]
35	rs2070744	NOS3	7	150690079	C	11.2%	9.69%	0.94 / 0.74	0.240	2012 Zhao[18]
36	rs1571942	PLXDC2	10	20542634	G	12.1%	8.53%	— / —	0.516	2011 Huang[1]
37	rs12219125	PLXDC2	10	20593087	T	12.1%	8.16%	1.00 / 1.00	0.975	2011 Huang[1]
38	rs4838605	ARHGAP22	10	49699957	C	9.7%	8.05%	— / —	0.381	2011 Huang[1]
39	rs11101355	ARHGAP22	10	49723037	T	11.2%	7.82%	— / 0.99	0.332	2011 Huang[1]
40	rs11101357	ARHGAP22	10	49723300	A	11.2%	7.82%	— / 0.99	0.332	2011 Huang[1]
41	rs4462262	MIR3924	10	59189178	T	5.8%	7.65%	1.00 / 0.98	0.155	2011 Huang[1]
42	rs7903146	TCF7L2	10	114758349	T	2.4%	3.07%	— / —	0.588	2015 Ding[19]
43	rs899036	API5	11	41682910	G	8.3%	11.05%	1.00 / 0.98	0.809	2010 Fu[4]; 2013 Zhang[20]

44	rs10501943	CNTN5	11	99946999	C	4.9%	2.72%	0.95 / 0.92	0.631	2010 Fu[4]
45	rs9565164	TBC1D4	13	76039376	C	22.8%	24.66%	— / —	0.479	2013 Sheu[6]
46	rs2031236	UCHL3	13	76169777	A	33.5%	29.42%	0.98 / 0.98	0.684	2013 Sheu[6]
47	rs7986566	COMMD6	13	76197931	C	33.5%	29.45%	1.00 / 0.99	0.708	2013 Sheu[6]
48	rs2038823	HS6ST3	13	96951433	T	7.3%	5.78%	— / —	0.293	2011 Huang[1]
49	rs3742872	PLEKHH1	14	68040838	A	4.9%	1.53%	— / 0.88	0.790	2012 Han[21]
50	rs10519765	FMN1	15	33205424	A	8.3%	9.69%	0.99 / —	0.874	2010 Fu[4]
51	rs832882	PLEKHO2	15	65150837	A	19.4%	17.01%	— / —	0.837	2012 Han[21]
52	rs1024611	CCL12	17	32579788	A	36.4%	44.22%	— / 0.98	0.909	2016 Wang[22]
53	rs599019	COLEC12	18	294495	C	41.8%	47.79%	1.00 / 0.96	0.841	2010 Fu[4]
54	rs13306430	ICAM1	19	10395624	A	0.0%	0.17%	0.41 / 0	0.977	2015 Fan[23]
55	rs5498	ICAM1	19	10395683	G	26.2%	26.46%	— / —	0.046	2015 Fan[23]
56	rs1800469	TGFB1	19	41860296	G	52.4%	45.92%	0.99 / 0.97	0.061	2014 Liu[24]
57	rs761207	JPH2	20	42758834	T	21.4%	19.56%	— / 0.96	0.405	2013 Huang[25]
58	rs6031415	JPH2	20	42769309	A	22.8%	17.69%	— / —	0.631	2013 Huang[25]

Abbreviations: chr., chromosome; MAF, minor allele frequency; HWE, Hardy-Weinberg equilibrium

[a]MAFs in Han Chinese from Beijing (CHB) (NCBI GRCh37.p13 assembly) from the online database of the dbSNP website (based on 1000 genomes project; https://www.ncbi.nlm.nih.gov/variation/tools/1000genomes/).

[b]The imputation quality (info) from IMPUTE v2 (http://mathgen.stats.ox.ac.uk/impute/impute_v2.html).

[c]Illumina HumanHap550-Duo BeadChips.

[d]Affymetrix-TWB chips.

References

1. Huang YC, Lin JM, Lin HJ, et al. Genome-wide association study of diabetic retinopathy in a Taiwanese population. Ophthalmology 2011;118:642-8.
2. Niu W, Qi Y. An updated meta-analysis of methylenetetrahydrofolate reductase gene 677C/T polymorphism with diabetic nephropathy and diabetic retinopathy. Diabetes research and clinical practice 2012;95:110-8.
3. Luo S, Wang F, Shi C, Wu Z. A Meta-Analysis of Association between Methylenetetrahydrofolate Reductase Gene (MTHFR) 677C/T Polymorphism and Diabetic Retinopathy. International journal of environmental research and public health 2016;13.
4. Fu YP, Hallman DM, Gonzalez VH, et al. Identification of Diabetic Retinopathy Genes through a Genome-Wide Association Study among Mexican-Americans from Starr County, Texas. Journal of ophthalmology 2010;2010.
5. Abhary S, Hewitt AW, Burdon KP, Craig JE. A systematic meta-analysis of genetic association studies for diabetic retinopathy. Diabetes 2009;58:2137-47.
6. Sheu WH, Kuo JZ, Lee IT, et al. Genome-wide association study in a Chinese population with diabetic retinopathy. Human molecular genetics 2013;22:3165-73.
7. Ma J, Li Y, Zhou F, Xu X, Guo G, Qu Y. Meta-analysis of association between the Pro12Ala polymorphism of the peroxisome proliferator-activated receptor-gamma2 gene and diabetic retinopathy in Caucasians and Asians. Molecular vision 2012;18:2352-60.
8. Meng N, Zhang Y, Li H, Ma J, Qu Y. Association of tumor necrosis factor alpha promoter polymorphism (TNF-alpha 238 G/A and TNF-alpha 308 G/A) with diabetic mellitus, diabetic retinopathy and diabetic nephropathy: a meta-analysis. Current eye research 2014;39:194-203.
9. Niu W, Qi Y, Wu Z, Liu Y, Zhu D, Jin W. A meta-analysis of receptor for advanced glycation end products gene: four well-evaluated polymorphisms with diabetes mellitus. Molecular and cellular endocrinology 2012;358:9-17.
10. Yuan D, Liu Q. Association of the receptor for advanced glycation end products gene polymorphisms with diabetic retinopathy in type 2 diabetes: a meta-analysis. Ophthalmologica Journal international d'ophtalmologie International journal of ophthalmology Zeitschrift fur Augenheilkunde 2012;227:223-32.
11. Lin HJ, Huang YC, Lin JM, Wu JY, Chen LA, Tsai FJ. Association of genes on chromosome 6, GRIK2 , TMEM217 and TMEM63B (linked to MRPL14)

with diabetic retinopathy. Ophthalmologica Journal international d'ophtalmologie International journal of ophthalmology Zeitschrift fur Augenheilkunde 2013;229:54-60.
12. Qiu M, Xiong W, Liao H, Li F. VEGF -634G>C polymorphism and diabetic retinopathy risk: a meta-analysis. Gene 2013;518:310-5.
13. Han L, Zhang L, Xing W, et al. The associations between VEGF gene polymorphisms and diabetic retinopathy susceptibility: a meta-analysis of 11 case-control studies. Journal of diabetes research 2014;2014:805801.
14. Zeng Y, Dai F, Yang K, Tang Y, Xu M, Zhou Y. Association between a vascular endothelial growth factor gene polymorphism (rs2146323) and diabetic retinopathy: a meta-analysis. BMC ophthalmology 2015;15:163.
15. Tian C, Fang S, Du X, Jia C. Association of the C47T polymorphism in SOD2 with diabetes mellitus and diabetic microvascular complications: a meta-analysis. Diabetologia 2011;54:803-11.
16. Hu C, Zhang R, Yu W, et al. CPVL/CHN2 genetic variant is associated with diabetic retinopathy in Chinese type 2 diabetic patients. Diabetes 2011;60:3085-9.
17. Hosseini SM, Boright AP, Sun L, et al. The association of previously reported polymorphisms for microvascular complications in a meta-analysis of diabetic retinopathy. Human genetics 2015;134:247-57.
18. Zhao S, Li T, Zheng B, Zheng Z. Nitric oxide synthase 3 (NOS3) 4b/a, T-786C and G894T polymorphisms in association with diabetic retinopathy susceptibility: A meta-analysis. Ophthalmic genetics 2012.
19. Ding Y, Hu Z, Yuan S, Xie P, Liu Q. Association between transcription factor 7-like 2 rs7903146 polymorphism and diabetic retinopathy in type 2 diabetes mellitus: A meta-analysis. Diabetes & vascular disease research 2015;12:436-44.
20. Zhang T, Pang C, Li N, Zhou E, Zhao K. Plasminogen activator inhibitor-1 4G/5G polymorphism and retinopathy risk in type 2 diabetes: a meta-analysis. BMC medicine 2013;11:1.
21. Han EC, Huang YC, Lin JM, et al. Association of the PLEKHO2 and PLEKHH1 gene polymorphisms with type 2 diabetic retinopathy in a Taiwanese population. Scienceasia 2012;38:340-8.
22. Wang W, He M, Huang W. Association of monocyte chemoattractant protein-1 gene 2518A/G polymorphism with diabetic retinopathy in type 2 diabetes mellitus: A meta-analysis. Diabetes research and clinical practice 2016;120:40-6.

23. Fan WY, Liu NP. Meta-analysis of association between K469E polymorphism of the ICAM-1 gene and retinopathy in type 2 diabetes. International journal of ophthalmology 2015;8:603-7.
24. Liu L, Jiao J, Wang Y, et al. TGF-beta1 gene polymorphism in association with diabetic retinopathy susceptibility: a systematic review and meta-analysis. PloS one 2014;9:e94160.
25. Huang YC, Lin HY, Lin HJ, et al. JPH2 is a novel susceptibility gene on chromosome 20q associated with diabetic retinopathy in a Taiwanese population. Scienceasia 2013;39:167-73.

Table S2 : 93 genetic SNPs were associated with DR ($p < 0.05$) under the additive model in Taiwanese population

No.	rs ID	Gene	Chr.	Position	Minor Allele	MAF (%) in CHB[a]	MAF (%) in controls	Info[b] (Chip1[c]/Chip2[d])	*P* for HWE in controls
1	rs60607614	MYSM1	1	59131088	G	18.90%	23.10%	0.89 / 0.87	0.625
2	rs1972588	LRP2	2	170052059	T	18.00%	20.24%	0.66 / 0.82	0.432
3	rs6718611	BBS5	2	170355378	A	17.00%	11.40%	0.99 / 1.00	0.916
4	rs6431331	SH3BP4	2	235940116	T	24.30%	26.70%	0.75 / —	0.076
5	rs35812816	PPARG	3	12439348	G	5.30%	5.10%	0.74 / 0.91	0.777
6	rs11931414	FSTL5	4	162363917	A	14.60%	14.80%	0.95 / —	0.260
7	rs17041044	FSTL5	4	162364557	T	14.60%	14.80%	0.95 / 1.00	0.260
8	rs13125474	FSTL5	4	162450029	T	8.30%	8.20%	0.96 / 0.96	0.112
9	rs34837397	FSTL5	4	162502717	T	6.30%	9.20%	0.74 / 0.77	0.079
10	rs4691013	FSTL5	4	162504877	G	17.50%	17.10%	0.75 / —	0.520
11	rs6852215	FSTL5	4	162518014	C	23.30%	22.60%	0.95 / 0.95	0.729
12	rs359147	FSTL5	4	162734835	T	34.00%	40.80%	— / —	0.054
13	rs13139950	FSTL5	4	162781322	G	48.10%	44.00%	0.92 / 0.93	0.120
14	rs76000640	FSTL5	4	162789702	T	21.80%	13.30%	0.72 / —	0.546
15	rs721197	FSTL5	4	162863837	A	17.50%	16.50%	0.99 / 1.00	0.672
16	rs9996797	FSTL5	4	162869274	A	17.50%	16.50%	0.99 / 1.00	0.672
17	rs80106981	FSTL5	4	162921481	A	31.60%	33.00%	0.95 / 0.97	0.065
18	rs62329628	FSTL5	4	162956556	A	23.80%	25.70%	0.96 / —	0.465
19	rs4691811	FSTL5	4	162993187	C	11.70%	12.20%	1.00 / 1.00	0.748

20	rs1388833	FSTL5	4	163000185	A	12.10%	12.20%	1.00 / 0.99	0.748
21	rs11100415	FSTL5	4	163070497	A	49.50%	49.50%	0.97 / 0.99	0.380
22	rs3756541	ITGA2	5	52303345	A	19.40%	15.48%	0.99 / 1.00	0.383
23	rs10942344	EDIL3	5	83490180	T	7.80%	7.80%	0.77 / 0.82	0.515
24	rs143520	CAMK4	5	110748363	C	9.70%	6.00%	0.92 / 0.96	0.965
25	rs766444	CAMK4	5	110822927	G	14.10%	9.60%	0.41 / 0.77	0.276
26	rs2776800	TMEM217	6	37188494	A	16.50%	23.20%	— / 0.58	0.690
27	rs865577	VEGFA	6	43742419	C	NA	37.41%	1.00 / 1.00	0.479
28	rs3024998	VEGFA	6	43745577	T	48.50%	37.80%	0.96 / —	0.443
29	rs3025017	VEGFA	6	43748357	A	10.70%	6.14%	0.47 / 0.53	0.915
30	rs3025018	VEGFA	6	43748795	T	NA	13.29%	0.51 / 0.54	0.118
31	rs17062031	GRIK2	6	101932545	C	7.80%	7.10%	0.94 / —	0.660
32	rs810197	GRIK2	6	102019977	C	9.70%	6.10%	0.98 / 0.94	0.362
33	rs2758334	SOD2	6	160110454	C	10.70%	12.93%	0.99 / 0.98	0.963
34	rs7782011	CHN2	7	29337601	G	47.60%	44.90%	0.97 / 0.94	0.264
35	rs6948298	CHN2	7	29397850	A	41.70%	37.20%	0.95 / —	0.487
36	rs10808275	AKR1B1	7	134132475	G	7.80%	10.70%	0.98 / 0.98	0.819
37	rs2974975	AKR1B1	7	134134135	G	6.80%	6.66%	0.52 / 0.58	0.509
38	rs73601616	PLXDC2	10	20126166	C	11.70%	8.70%	0.98 / 0.96	0.372
39	rs11594168	PLXDC2	10	20137692	C	7.80%	6.00%	0.56 / —	0.965
40	rs12766994	PLXDC2	10	20140808	C	47.10%	42.30%	— / 0.97	0.947
41	rs11596700	PLXDC2	10	20144680	A	43.20%	37.90%	0.96 / —	0.416
42	rs7088396	PLXDC2	10	20276683	C	49.50%	54.10%	0.57 / 0.55	0.797

43	rs4748635	PLXDC2	10	20363843	T	31.60%	29.40%	0.99 / 0.98	0.877
44	rs143997976	PLXDC2	10	20415355	C	NA	37.90%	0.93 / 0.93	0.750
45	rs4431920	PLXDC2	10	20424697	C	49.00%	48.00%	0.98 / 0.98	0.748
46	rs4255445	PLXDC2	10	20436401	T	48.50%	48.10%	1.00 / 0.99	0.797
47	rs4748644	PLXDC2	10	20493590	C	48.50%	52.20%	0.99 / 0.99	0.462
48	rs7073942	PLXDC2	10	20527593	A	43.70%	35.70%	0.93 / 0.92	0.405
49	rs11011909	PLXDC2	10	20540889	G	12.10%	8.50%	0.99 / 1.00	0.512
50	rs4838606	ARHGAP22	10	49700429	T	11.70%	8.20%	0.98 / —	0.418
51	rs10776609	ARHGAP22	10	49703048	C	34.00%	35.40%	0.98 / 0.97	0.840
52	rs7910353	ARHGAP22	10	49719217	T	12.10%	8.30%	0.96 / 0.99	0.464
53	rs11101385	ARHGAP22	10	49771150	A	13.10%	6.60%	0.87 / —	0.506
54	rs10857599	ARHGAP22	10	49781242	C	10.70%	7.80%	0.97 / 0.97	0.332
55	rs59419235	ARHGAP22	10	49783358	G	33.50%	29.80%	0.96 / 0.94	0.569
56	rs1445164	ARHGAP22	10	49800427	G	43.20%	41.30%	0.83 / —	0.254
57	rs73302224	ARHGAP22	10	49801412	C	20.90%	15.90%	0.68 / 0.69	0.054
58	rs78203857	TCF7L2	10	114830372	G	9.70%	8.70%	0.53 / —	0.872
59	rs61893374	CNTN5	11	98991499	T	11.70%	6.20%	0.54 / —	0.261
60	rs75241175	CNTN5	11	99187128	G	11.20%	6.70%	0.71 / —	0.779
61	rs7104324	CNTN5	11	99604914	T	14.10%	18.50%	0.66 / —	0.458
62	rs10894143	CNTN5	11	99777082	C	19.90%	21.10%	0.99 / 0.99	0.979
63	rs140793340	CNTN5	11	99909277	A	11.70%	6.20%	0.46 / 0.68	0.259
64	rs72998878	CNTN5	11	99979223	C	12.60%	16.70%	0.87 / —	0.735
65	rs2112071	CNTN5	11	99999475	A	48.10%	39.70%	— / —	0.611

66	rs78714603	CNTN5	11	100216825	G	4.90%	5.40%	0.67 / 0.68	0.324
67	rs1431833	TBC1D4	13	75870947	A	5.80%	13.10%	0.98 / 0.98	0.130
68	rs1469600	TBC1D4	13	75895847	T	30.60%	38.90%	0.97 / 0.97	0.106
69	rs77971785	TBC1D4	13	75976317	A	14.10%	7.40%	0.51/ —	0.614
70	rs517984	HS6ST3	13	97288935	C	13.10%	13.60%	0.98 / —	0.826
71	rs16953570	HS6ST3	13	97438801	T	26.70%	34.00%	— / —	0.792
72	rs182127416	HS6ST3	13	97452220	A	NA	32.70%	0.98 / 0.97	0.534
73	rs11840842	HS6ST3	13	97460000	C	37.40%	42.50%	0.93 / 0.94	0.453
74	rs62001318	FMN1	15	33167503	G	27.70%	32.65%	0.96 / 0.99	0.862
75	rs11631278	FMN1	15	33214070	T	5.80%	7.50%	0.97 / 0.95	0.766
76	rs12903814	FMN1	15	33449902	A	6.30%	8.80%	0.85 / 0.98	0.829
77	rs71462852	FMN1	15	33453557	A	6.30%	8.70%	0.85 / —	0.876
78	rs4780089	FMN1	15	33459488	G	45.60%	41.70%	0.98 / 0.98	0.234
79	rs142644390	FMN1	15	33474150	G	11.20%	14.80%	0.84 / 0.92	0.840
80	rs621636	COLEC12	18	322522	T	33.50%	38.60%	— / 0.99	0.152
81	rs34039285	COLEC12	18	325455	G	34.90%	29.60%	0.53 / —	0.096
82	rs505110	COLEC12	18	331506	G	43.70%	41.50%	— / 0.89	0.880
83	rs7233716	COLEC12	18	383047	G	25.70%	28.20%	1.00 / 0.99	0.064
84	rs6506133	COLEC12	18	387120	A	27.70%	28.20%	— / 0.99	0.118
85	rs12454569	COLEC12	18	394366	T	24.80%	25.30%	0.97 / —	0.232
86	rs117029442	COLEC12	18	405037	A	34.50%	32.80%	1.00 / 1.00	0.538
87	rs1940436	COLEC12	18	415401	T	45.60%	41.70%	0.99 / 1.00	0.342
88	rs8099806	COLEC12	18	458860	G	50.00%	55.61%	0.95 / 0.98	0.468

89	rs73358562	COLEC12	18	482735	A	30.10%	29.25%	0.95 / 0.98	0.744
90	rs9789175	COLEC12	18	494878	C	30.60%	28.20%	0.58 / —	0.177
91	rs9962486	COLEC12	18	499239	G	25.70%	25.40%	0.54 / —	0.468
92	rs75894078	JPH2	20	42745947	A	11.70%	14.50%	0.93 / 0.93	0.312
93	rs139772112	JPH2	20	42754812	G	NA	9.11%	0.60 / 0.64	0.770

Abbreviations: chr., chromosome; MAF, minor allele frequency; HWE, Hardy-Weinberg equilibrium

[a] MAFs in Han Chinese from Beijing (CHB) (NCBI GRCh37.p13 assembly) from the online database of the dbSNP website (based on 1000 genomes project; https://www.ncbi.nlm.nih.gov/variation/tools/1000genomes/).

[b]The imputation quality (info) from IMPUTE v2 (http://mathgen.stats.ox.ac.uk/impute/impute_v2.html).

[c]Illumina HumanHap550-Duo BeadChips. [d]Affymetrix-TWB chips.

Table S3. Relationship between total risk score and the risk of diabetic retinopathy in the derivation, test and validation samples

Risk Score	Overall Sample	Derivation Sample	Test Sample	Validation Sample
**No of subjects (%)[a]**				
**All categories**				
< 0.5	132 (12.9)	35 (5.7)	56 (19.6)	7 (0.0)
0.5-1.5	233 (25.8)	104 (21.2)	202 (30.2)	354 (13.3)
1.5-2.5	344 (50.9)	136 (39.7)	111 (59.5)	150 (26.7)
2.5-3.5	203 (67.5)	126 (55.6)	116 (72.4)	15 (33.3)
> 3.5	65 (86.2)	91 (82.4)	-	-
**2 categories**				
< 2.28	627 (32.2)	234 (23.9)	367 (37.3)	494 (16.6)
≧2.28	350 (69.4)	258 (64.7)	118 (72.0)	32 (31.3)
**Range**	0.10 ~ 3.88	0.10 ~ 4.47	0.10 ~ 3.45	0.42 ~ 2.94

[a] Numbers indicate subjects in each risk score category; percentages indicate the percentage of DR cases in each risk category. Risk score is based on the full model including, age, diabetic duration, HbA1c, systolic blood pressure and genetic risk score which combined with four genes.

Table S4. Linkage disequilibrium coefficients (D' and r^2) of the SNPs

No	SNP	Gene	Chr	Position (GRCh37)	D'	R^2
1	rs4748644	PLXDC2	10	20493590	1	1
2	rs1571942	PLXDC2	10	20542634	0.588	0.045
3	rs12219125	PLXDC2	10	20593087	0.588	0.045
4	rs11101385	ARHGAP22	10	49771150	1	1
5	rs11101355	ARHGAP22	10	49723037	0.8	0.533
6	rs11101357	ARHGAP22	10	49723300	0.8	0.533
7	rs4838605	ARHGAP22	10	49699957	0.712	0.362
8	rs61893374	CNTN5	11	98991499	1	1
9	rs10501943	CNTN5	11	99946999	1	0.007
10	rs142644390	FMN1	15	33474150	1	1
11	rs10519765	FMN1	15	33205424	0.476	0.003

Abbreviations: SNP, single nucleotide polymorphisms; chr., chromosome;

Table S5_1. Comparison of demographics characteristic of two databases for derivation set and test set

	Derivation Sample (N = 528)	Test Sample (N = 527)	*P* value
**Gender**			0.879
Male	276 (52.3%)	273 (51.8%)	
Female	252 (47.7%)	254 (48.2%)	
**Age (years)**			0.147
< 55	175 (33.1)	154 (29.2)	
55-65	199 (37.7)	191(36.2)	
> 65	154 (29.2)	182 (34.5)	
**DM duration (years)**			0.688
≦10	302 (57.7)	295 (56.5)	
> 10	221 (42.3)	227 (43.5)	
**Age of onset (years)**			0.812
< 45	172 (32.9)	162 (31.0)	
45-55	200 (38.2)	206 (39.5)	
> 55	151 (28.9)	154 (29.5)	
**HbA1c**			0.436
≦8	321 (60.8)	308 (58.4)	
> 8	207 (39.2)	219 (41.6)	
**SBP**			0.976
< 140	248 (49.6)	245 (49.7	
≧ 140	252 (50.4)	248 (50.3)	
**DBP**			0.950
< 90	411 (82.2)	406 (82.4)	
≧ 90	89 (17.8)	87 (17.6)	
**Fasting glucose**[#]			0.808
< 126	142 (33.9)	155 (35.7)	
126-155	141 (33.7)	146 (33.6)	
> 155	136 (32.5)	133 (30.6)	
**HDL**[#]			0.862
< 41	126 (33.4)	131 (35.3)	
41-52	126 (33.4)	120 (32.3)	
> 52	125 (33.2)	120 (32.3)	
**LDL**[#]			0.520
< 103	127 (33.7)	139 (37.4)	
103-132	129 (34.2)	125 (33.6)	

> 132	121 (32.1)	108 (29.0)	
**TG**#			0.630
< 103	126 (33.7)	113 (30.5)	
103-171	124 (33.2)	127 (34.2)	
> 171	124 (33.2)	131 (35.3)	
**eGFR**#			0.712
> 90	222 (58.9)	230 (61.8)	
60-90	112 (29.7)	103 (27.7)	
< 60	43 (11.4)	39 (10.5)	
**ACR**#			0.655
< 30	236 (64.5)	230 (63.9)	
30-300	99 (27.0)	105 (29.2)	
> 300	31 (8.5)	25 (6.9)	

Values are presented as N (%).

Abbreviation: T2D, type 2 diabetes; DR, diabetic retinopathy; DM duration: diabetes mellitus duration; HbA1c: hemoglobin A1c; SBP/DBP: systolic/diastolic blood pressure; HDL: high density lipoprotein; LDL: low density lipoprotein; TG: triglyceride; eGFR: estimated glomerular filtration rate; ACR: urine albumin creatinine ratio.

P value for chi square test.

#Results from limited subjects (N=366 and 360 for model building and internal validation, respectively).

Table S5_2. Demographic characteristic of the study population for derivation set

	T2D control (N = 294)	DR (N = 234)	OR	*P* value
**Gender**				
**Male**	156 (53.1%)	120 (51.3%)	Ref.	Ref.
**Female**	138 (46.9%)	114 (48.7%)	1.07	0.684
**Age (years)**				
< 55	119 (40.5)	56 (23.9)	Ref.	Ref.
55-65	95 (32.3)	104 (44.4)	2.33	<0.001*
> 65	80 (27.2)	74 (31.6)	1.97	0.003*
**DM duration (years)**				
≦10	210 (71.4)	92 (40.2)	Ref.	Ref.
> 10	84 (28.6)	137 (59.8)	3.72	<0.001*
**Age of onset (years)**				
< 45	88 (29.9)	84 (36.7)	Ref.	Ref.
45-55	108 (36.7)	92 (40.2)	0.89	0.585
> 55	98 (33.3)	53 (23.1)	0.57	0.013*
**HbA1c**				
≦8	198 (67.3)	123 (52.6)	Ref.	Ref.
> 8	96 (32.7)	111 (47.4)	1.86	0.001*
**SBP**				
< 140	161 (59.2)	87 (38.2)	Ref.	Ref.
≧140	111 (40.8)	141 (61.8)	2.35	<0.001*
**DBP**				
< 90	236 (86.8)	175 (76.8)	Ref.	Ref.
≧90	36 (13.2)	53 (23.2)	1.96	0.004*
**Fasting glucose**[#]				
< 126	102 (34.7)	40 (32.0)	Ref.	Ref.
126-155	108 (36.7)	33 (26.4)	0.78	0.360
> 155	84 (28.6)	52 (41.6)	1.58	0.075
**HDL**[#]				
< 41	95 (32.6)	31 (36.0)	Ref.	Ref.
41-52	98 (33.7)	28 (32.6)	0.88	0.655
> 52	98 (33.7)	27 (31.4)	0.84	0.573
**LDL**[#]				
< 103	94 (32.3)	33 (38.4)	Ref.	Ref.
103-132	101 (34.7)	28 (32.6)	0.79	0.422

> 132	96 (33.0)	25 (29.1)	0.74	0.323
**TG[#]**				
< 103	98 (33.9)	28 (32.9)	Ref.	Ref.
103-171	92 (31.8)	32 (37.6)	1.22	0.507
> 171	99 (34.3)	25 (29.4)	0.88	0.690
**eGFR[#]**				
> 90	182 (62.5%)	40 (46.5%)	Ref.	Ref.
60-90	82 (28.2%)	30 (34.9%)	1.67	0.065
< 60	27 (9.3%)	16 (18.6%)	2.70	0.006*
**ACR[#]**				
< 30	203 (70.7%)	33 (41.8%)	Ref.	Ref.
30-300	68 (23.7%)	31 (39.2%)	2.80	<0.001*
> 300	16 (5.6%)	15 (19.0%)	5.77	<0.001*

Values are presented as N (%).

Abbreviation: T2D, type 2 diabetes; DR, diabetic retinopathy; DM duration: diabetes mellitus duration; HbA1c: hemoglobin A1c; SBP/DBP: systolic/diastolic blood pressure; HDL: high density lipoprotein; LDL: low density lipoprotein; TG: triglyceride; eGFR: estimated glomerular filtration rate; ACR: urine albumin creatinine ratio; Ref., reference.

[#]Results from limited subjects (N=287 and 79 for T2D control and DR, respectively).

* represent *P* value less than 0.05.

Table S5_3. Demographic characteristics of the study population for test set

	T2D control (N = 293)	DR (N = 234)	OR	*P* value
**Gender**				
**Male**	155 (52.9%)	118 (50.4%)	Ref.	Ref.
**Female**	138 (47.1%)	116 (49.6%)	1.10	0.572
**Age (years)**				
< 55	110 (37.5)	44 (18.8)	Ref.	Ref.
55-65	105 (35.8)	104 (44.4)	2.47	<0.001*
> 65	78 (26.6)	86 (36.8)	2.73	<0.001*
**DM duration (years)**				
≦10	210 (71.7)	85 (37.1)	Ref.	Ref.
> 10	83 (28.3)	144 (62.9)	4.29	<0.001*
**Age of onset (years)**				
< 45	73 (24.9)	89 (38.9)	Ref.	Ref.
45-55	126 (43.0)	80 (34.9)	0.52	0.002*
> 55	94 (32.1)	60 (26.2)	0.52	0.005*
**HbA1c**				
≦8	197 (67.2)	111 (47.4)	Ref.	Ref.
> 8	96 (32.8)	123 (52.6)	2.27	<0.001*
**SBP**				
< 140	155 (58.5)	90 (39.5)	Ref.	Ref.
≧ 140	110 (41.5)	138 (60.5)	2.16	<0.001*
**DBP**				
< 90	223 (84.2)	183 (80.3)	Ref.	Ref.
≧ 90	42 (15.8)	45 (19.7)	1.31	0.260
**Fasting glucose**$^{\#}$				
< 126	100 (34.7)	55 (37.7)	Ref.	Ref.
126-155	108 (37.5)	38 (26.0)	0.64	0.077
> 155	80 (27.8)	53 (36.3)	1.21	0.446
**HDL**$^{\#}$				
< 41	103 (36.4)	28 (31.8)	Ref.	Ref.
41-52	96 (33.9)	24 (27.7)	0.92	0.788
> 52	84 (29.7)	36 (40.9)	1.58	0.119
**LDL**$^{\#}$				
< 103	110 (38.7)	29 (33.0)	Ref.	Ref.
103-132	88 (31.0)	37 (42.0)	1.60	0.103
> 132	86 (30.3)	22 (25.0)	0.97	0.924

**TG#**				
< 103	84 (29.7)	29 (33.0)	Ref.	Ref.
103-171	102 (36.0)	25 (28.4)	0.71	0.269
> 171	97 (34.3)	34 (38.6)	1.02	0.959
**eGFR#**				
> 90	190 (66.9%)	40 (45.5%)	Ref.	Ref.
60-90	72 (25.4%)	31 (35.2%)	2.05	0.010*
< 60	22 (7.7%)	17 (19.3%)	3.67	<0.001*
**ACR#**				
< 30	195 (70.4%)	35 (42.2%)	Ref.	Ref.
30-300	67 (24.2%)	38 (45.8%)	3.16	<0.001*
> 300	15 (5.4%)	10 (12.0%)	3.71	0.003*

Values are presented as N (%).

Abbreviation: T2D, type 2 diabetes; DR, diabetic retinopathy; DM duration: diabetes mellitus duration; HbA1c: hemoglobin A1c; SBP/DBP: systolic/diastolic blood pressure; HDL: high density lipoprotein; LDL: low density lipoprotein; TG: triglyceride; eGFR: estimated glomerular filtration rate; ACR: urine albumin creatinine ratio; Ref., reference.

#Results from limited subjects (N=277 and 83 for T2D control and DR, respectively).

* represent *P* value less than 0.05.

Table S6. Demographics of the study population for external validation

	T2D control (N = 447)	DR (N = 95)	OR	*P* value
**Gender**				
**Male**	244 (54.6%)	46 (48.4%)	Ref.	Ref.
**Female**	203 (45.4%)	49 (51.6%)	1.28	0.275
**Age (years)**				
< 55	97 (21.7)	15 (15.8)	Ref.	Ref.
55-65	161 (36.0)	33 (34.7)	1.33	0.403
> 65	189 (42.3)	47 (49.5)	1.61	0.140
**DM duration (years)**				
≦10	315 (70.5)	59 (62.1)	Ref.	Ref.
> 10	132 (29.5)	36 (37.9)	1.46	0.111
**Age of onset (years)**				
< 45	77 (17.2)	21 (22.1)	Ref.	Ref.
45-55	150 (33.6)	36 (37.9)	0.88	0.678
> 55	220 (49.2)	38 (40.0)	0.63	0.131
**HbA1c**				
≦8	351 (78.5)	53 (55.8)	Ref.	Ref.
> 8	96 (21.5)	42 (44.2)	2.90	<0.001*
**SBP**				
< 140	287 (66.0)	50 (54.3)	Ref.	Ref.
≧ 140	148 (34.0)	42 (45.7)	1.63	0.036*
**DBP**				
< 90	389 (89.4)	86 (93.5)	Ref.	Ref.
≧ 90	46 (10.6)	6 (6.5)	0.59	0.241
**Fasting glucose**#				
< 126	211 (47.7)	28 (30.8)	Ref.	Ref.
126-155	119 (26.9)	19 (20.9)	1.20	0.561
> 155	112 (25.3)	44 (48.4)	2.96	<0.001*
**HDL**#				
< 41	81 (33.9)	6 (15.8)	Ref.	Ref.
41-52	83 (34.7)	17 (44.7)	2.77	0.042*
> 52	75 (31.4)	15 (39.5)	2.70	0.051
**LDL**#				
< 103	210 (57.7)	13 (59.1)	Ref.	Ref.
103-132	86 (23.6)	5 (22.7)	0.94	0.908
> 132	68 (18.7)	4 (18.2)	0.95	0.931

**TG**#				
< 103	161 (40.8)	19 (42.2)	Ref.	Ref.
103-171	120 (30.4)	15 (33.3)	1.06	0.875
> 171	114 (28.9)	11 (24.4)	0.82	0.613
**eGFR**#				
> 90	134 (30.9%)	10 (11.0%)	Ref.	Ref.
60-90	144 (33.2%)	37 (40.7%)	3.44	0.001*
< 60	156 (35.9%)	44 (48.4%)	3.78	<0.001*.
**ACR**#				
< 30	197 (48.3%)	13 (39.4%)	Ref.	Ref.
30-300	114 (27.9%)	14 (42.4%)	1.86	0.123
> 300	97 (23.8%)	6 (18.2%)	0.94	0.899

Values are presented as N (%).

Abbreviation: T2D, type 2 diabetes; DR, diabetic retinopathy; DM duration: diabetes mellitus duration; HbA1c: hemoglobin A1c; SBP/DBP: systolic/diastolic blood pressure; HDL: high density lipoprotein; LDL: low density lipoprotein; TG: triglyceride; eGFR: estimated glomerular filtration rate; ACR: urine albumin creatinine ratio; Ref., reference.

#Results from limited subjects (N=239 and 38 for T2D control and DR, respectively).

* represent *P* value less than 0.05.

Table S7. The sample size was calculated for a type 1 error of 5% and a power of 80%.

		OR=1.5	OR=2.0	OR=2.5	OR=3.0
			**Sample Size (N)**		
Y[a]=0.2	x=0.2	1623	**511**	277	186
	x=0.3	1250	397	216	145
	x=0.4	1104	352	193	130
	x=0.5	1069	343	188	127
Y=0.5	x=0.2	1213	428	254	183
	x=0.3	924	326	193	139
	x=0.4	808	285	168	121
	x[b]=0.5	775	272	161	115

G*power Version 3.1.7 was used for sample size calculation.

a. The prevalence of dependent variable (Y, DR status)

b. The prevalence of independent variable (X)

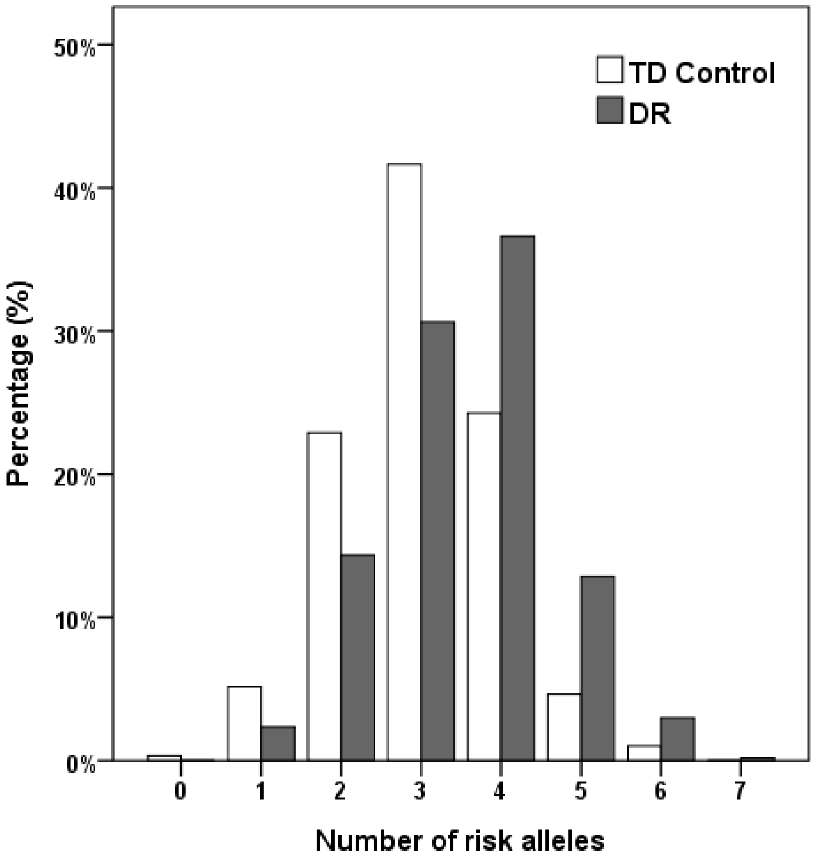

Figure S1. Distribution of the number of risk alleles.

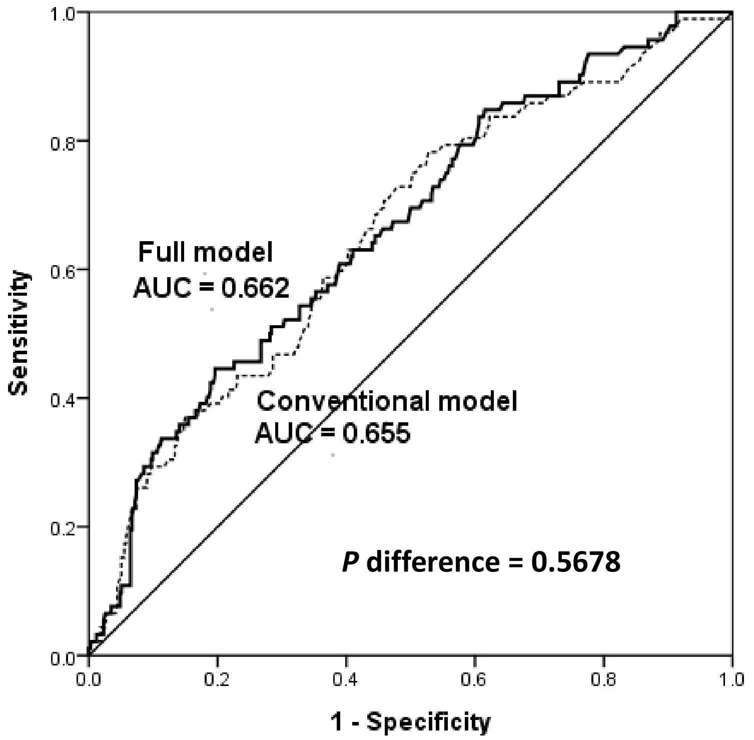

Figure S2. Receiver Operating Characteristic (ROC) curve and area under the curve (AUC) for validation samples. The predictive ability of the best-fit and conventional models. ROC curve and AUC for the "best-fit" (solid line; AUC = 0.662) and "conventional" (dotted line; AUC = 0.655) models. The diagonal line indicates zero predictive value of the model.

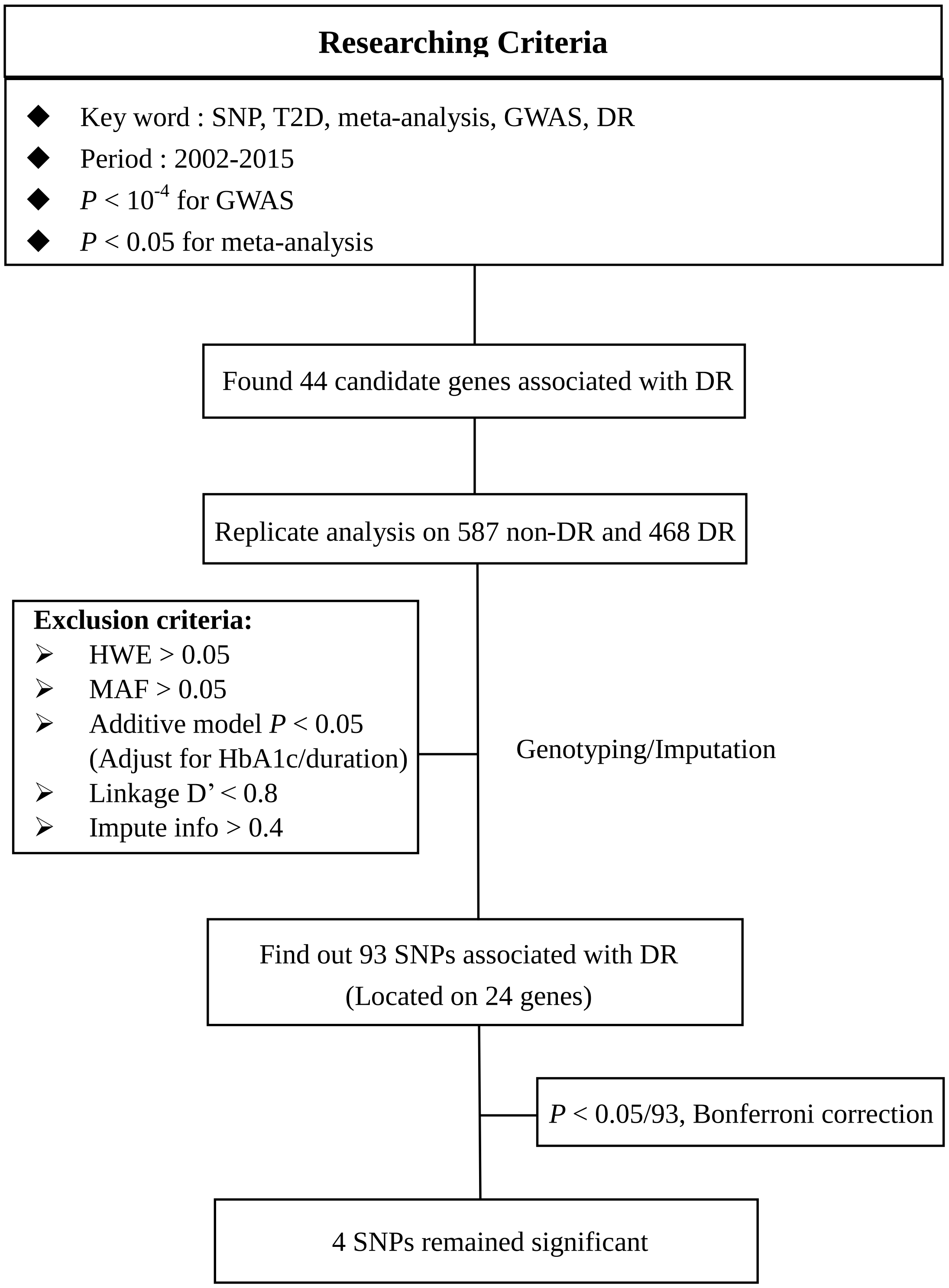

Figure S3. Flow chart for selecting genetic markers into genetic risk score
